# Difference of clinical features in childhood *Mycoplasma pneumoniae *pneumonia

**DOI:** 10.1186/1471-2431-10-48

**Published:** 2010-07-06

**Authors:** You-Sook Youn, Kyung-Yil Lee, Ja-Young Hwang, Jung-Woo Rhim, Jin-Han Kang, Joon-Sung Lee, Ji-Chang Kim

**Affiliations:** 1 Department of Pediatrics, College of Medicine, The Catholic University of Korea, Seoul, Korea; 2 Department of Radiology, College of Medicine, The Catholic University of Korea, Seoul, Korea

## Abstract

**Background:**

*M. pneumoniae *pneumonia (MP) has been reported in 10-40% of community-acquired pneumonia cases. We aimed to evaluate the difference of clinical features in children with MP, according to their age and chest radiographic patterns.

**Methods:**

The diagnosis of MP was made by examinations at both admission and discharge and by two serologic tests: the indirect microparticle agglutinin assay (≥1:40) and the cold agglutinins titer (≥1:32). A total of 191 children with MP were grouped by age: ≤2 years of age (29 patients), 3-5 years of age (81 patients), and ≥6 years of age (81 patients). They were also grouped by pneumonia pattern: bronchopneumonia group (96 patients) and segmental/lobar pneumonia group (95 patients).

**Results:**

Eighty-six patients (45%) were seroconverters, and the others showed increased antibody titers during hospitalization. Among the three age groups, the oldest children showed the longest duration of fever, highest C-reactive protein (CRP) values, and the most severe pneumonia pattern. The patients with segmental/lobar pneumonia were older and had longer fever duration and lower white blood cell (WBC) and lymphocyte counts, compared with those with bronchopneumonia. The patient group with the most severe pulmonary lesions had the most prolonged fever, highest CRP, highest rate of seroconverters, and lowest lymphocyte counts. Thrombocytosis was observed in 8% of patients at admission, but in 33% of patients at discharge.

**Conclusions:**

In MP, older children had more prolonged fever and more severe pulmonary lesions. The severity of pulmonary lesions was associated with the absence of diagnostic IgM antibodies at presentation and lymphocyte count. Short-term paired IgM serologic test may be mandatory for early and definitive diagnosis of MP.

## Background

*Mycoplasma pneumoniae *(*M. pneumoniae*) is an important causative organism of respiratory infections in children and young adults. *M. pneumoniae *pneumonia (MP) has been reported in 10-40% of community-acquired pneumonia cases, and recent studies have indicated that younger children (<5 years of age), as well as school-aged children, are prone to *M. pneumoniae *infection [1-6]. In Korea, *M. pneumoniae *epidemics have occurred every 3-4 years since the 1980s; in the most recent epidemics, the peak age was younger than that seen previously [6]. However, few studies have attempted a clinical comparison of MP according to age [4,5].

Difficulties exist in the detection of etiologic agents, including *M. pneumoniae *for lower respiratory tract infections in children (especially younger children) with regard to adequate sampling of respiratory materials for pathogen culture and polymerase chain reaction (PCR), and the need for paired blood sampling for serologic tests. In addition, it is known that in some patients, the diagnostic antibodies are not detected in the early stage of *M. pneumoniae *infection [1].

Although *M. pneumoniae *is a small bacterium that can induce pneumonia, the immunopathogenesis of this agent in humans is poorly understood. Clinical and experimental studies support the hypothesis that lung injury in *M. pneumoniae *infections is associated with the cell-mediated immunity of the host [7-10], including temporary anergy of purified protein derivatives (PPD) [9] and the dramatic beneficial effect of corticosteroids on severe MP in adults and children [7,10-13]. Therefore, it is expected that the severity of pulmonary lesions in MP might differ with the age of the patients, and that laboratory findings might differ according to the severity of pneumonia.

In the present study, we used two IgM serologic tests and two examinations at admission and discharge to characterize the clinical features, laboratory findings, and chest radiographic findings in children with MP during a recent epidemic in South Korea.

## Methods

We retrospectively analyzed the medical records and chest radiographic findings of 191 children with MP who were admitted to The Catholic University of Korea, Daejeon St. Mary's Hospital during a nationwide MP epidemic, from January 2006 through December 2007. A total of 1,083 patients with pneumonia or lower respiratory tract infections were admitted during this period. Among them, we selected patients with MP using two IgM serologic tests: the indirect microparticle agglutinin assay (MAA: Serodia-Myco II, Fujirebio, Japan; positive cutoff value ≥1:40) and the cold agglutinins titer (positive cutoff value ≥1:32). Following parental consent, both assays and some laboratory indices were routinely performed twice: once at the time of admission and once at discharge (mean: 6.0 ± 2.1 days apart). Subjects were selected for inclusion in the study if seroconversion was shown on both assays during admission, or if increased MAA-positive titers (≥4-fold) with corresponding cold agglutinin titers (including seroconversion) were displayed on the second test. Patients who tested positive in both assays at admission, but did not have increased or decreased titers at discharge, were regarded as having recent past infection and were excluded from the study (38 cases). Blood culture for bacterial pathogens was performed for all pneumonia patients. Nasopharyngeal aspirates or sputum for viral agents (influenza viruses type A and B, parainfluenza viruses, respiratory syncytial virus, and adenoviruses) and PCR for *M. pneumoniae *were examined in the majority of patients (129 patients).

The chest radiographic patterns at admission of patients with MP were divided into two groups. Patients with increased nodular densities along the bronchial trees and/or an interstitial pattern on the unilateral or bilateral lung fields were designated as the bronchopneumonia group. Patients with distinctive subsegmental, segmental or lobar consolidation were designated as the segmental/lobar pneumonia group. The chest radiographic findings were reviewed and classified independently by two pediatricians (KY Lee and YS Youn) and one pediatric radiologist (JC Kim).

We divided the 191 children with MP into three groups according to age: ≤2 years of age (29 patients), 3-5 years of age (81 patients), and ≥6 years of age (81 patients), and into another two groups according to pneumonia pattern: the bronchopneumonia group (96 cases) and the segmental/lobar pneumonia group (95 cases). In addition, the children aged ≥6 years (81 patients) were classified into three groups based on the severity of pneumonia: the bronchopneumonia group (25 patients), the mild segmental/lobar group (33 patients), and the severe segmental/lobar group (23 patients). The mild segmental/lobar pattern was defined as having an area of consolidation in less than one lobe without pleural effusion, while the severe segmental/lobar pattern was defined as having an area of consolidation over one lobe, including multiple lobe involvement and/or any consolidation with pleural effusion.

We also evaluated the pneumonia patients according to diagnostic antibody status. We compared the clinical and laboratory characteristics among the groups. The study was approved by our Institutional Review Board.

### Statistical analysis

Statistical analyses were performed using the Statistical Package for the Social Science for Windows version 12.0 (SPSS, Chicago, IL, USA). Continuous variables are reported as the mean ± standard deviation. Statistical significance was assessed using the χ^2 ^test for categorical variables, and the independent sample *t*-test, paired *t*-test, and one-way analysis of variation (ANOVA) for continuous variables. A *p *value < 0; 0.05 was considered statistically significant.

## Results

### Clinical and laboratory characteristics of *M. pneumoniae *pneumonia according to age

**Figure 1:**
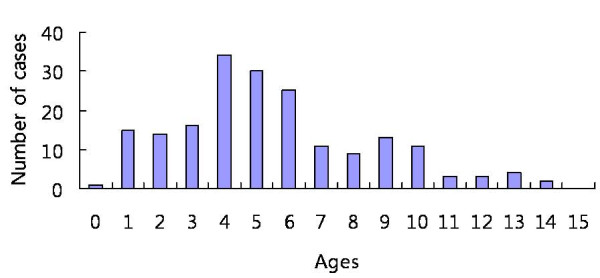
**Age distribution of our patients**.

The mean age of the subjects was 5.5 ± 3.0 years (range, 9 months-14 years), and the male-to-female ratio was 1:1.1. The age distribution of the patients is shown in figure 1. The patients had symptoms and signs indicative of pneumonia at the time of admission. All patients had a fever (>38°C per axilla) and cough, and the majority of patients had abnormal breath sounds on auscultation. Of the 191 patients with MP, 86 were seroconverters (i.e., IgM-negative at admission to IgM-positive at discharge) for both assays, and 105 seropositive patients showed increased MAA titers (>4-fold) with corresponding cold agglutinin titers during hospitalization. The median titers of MAA and cold agglutinins in seroconverters at discharge were 1:160 (range, 1:40-1:2,560) and 1:32 (range, 1:16-1:512), respectively. The median titers of both assays in seropositive patients were 1:160 (range, 1:40-1:640) and 1:16 (range, 1:1-1:512) at admission, and 1:640 (range, 1:80-1:10,240) and 1:64 (range, 1:8-1:1,024) at discharge, respectively. PCR assay for *M. pneumoniae *was performed in 129 patients; 37 patients (28.7%) were positive. No patient showed blood culture positive for bacterial pathogens including *Streptococcus pneumoniae*. A viral study performed in the same 129 patients revealed that 2 patients were co-infected with respiratory viruses (respiratory syncytial virus and parainfluenza virus A). Extrapulmonary manifestations of *M. pneumoniae *were observed as 19 cases of skin rash, 9 cases of abnormal hepatic enzymes (AST and ALT, >2-fold of normal values), and 1 case of encephalopathy.

We observed significant differences in certain parameters among the three age groups (Table 1). The total duration of fever tended to be longer, and the frequency of segmental/lobar pneumonia was significantly higher in patients ≥6 years of age compared with the younger groups (69.1% vs. 40.7% vs. 20.7%, respectively, *p *< 0.001). The patients who had fever lasting ≥7 days were also more strongly affiliated with the older groups (*p *= 0.04). Laboratory findings revealed that white blood cell (WBC) count, lymphocyte differential, and platelet count were lower in the group of patients aged ≥6 years; however, C-reactive protein (CRP) values were higher in this group (Table 1). Compared with at admission, at discharge the MP patients had significantly increased levels of lymphocyte differential (44.3% vs. 28.8%, respectively; *p *= 0.001), total IgG (921 vs. 893 mg/dL, respectively; *p *= 0.01) and platelet count (371,000/μL vs. 262,000/μL, respectively; *p *< 0.001) (data not shown).

**Table 1:** Clinical and laboratory findings according to age

Age groups	≤2 y	3-5 y	≥6 y	*P*
	(n = 29)	(n = 81)	(n = 81)	
Clinical characteristics				
Age (y)	2.0 ± 0.7	4.2 ± 0.8	8.3 ± 2.3	<0.001
Duration of fever (d)				
Before admission	3.1 ± 2.3	3.6 ± 2.8	4.2 ± 2.3	0.09
Total duration	4.8 ± 2.9	5.4 ± 3.3	6.1 ± 2.9*	0.1
Cases of ≥7 d (n,%) ^†^	9 (31)	29 (36)	40 (49)	0.04
Hospitalization (d)	6.9 ± 1.8	6.9 ± 2.3	6.8 ± 2.1	0.9
Pneumonia (n,%)				
Bronchopneumonia	23 (79)	48 (59)	25 (31)	<0.001
Segmental/lobar	6 (21)	33 (41)	56 (69)	<0.001
				
Laboratory findings				
Hemoglobin (g/dL)	12.2 ± 0.9	12.1 ± 0.9	12.3 ± 0.9	0.6
Leukocyte (×10^3^//μL)	11.2 ± 7.3	7.9 ± 3.5	7.1 ± 2.40	<0.001
Neutrophil (%)	47 ± 14	61 ± 14	65 ± 11	<0.001
Lymphocyte (%)	42 ± 13	29 ± 13	24 ± 9	<0.001
Monocyte (%)	9 ± 3	8 ± 3	8 ± 3	0.2
Platelet (×10^3^/μL)	319 ± 111	266 ± 91	251 ± 75	0.002
CRP (mg/dL)	1.6 ± 2.9	3.8 ± 4.8	4.1 ± 4.1	0.03
ESR (mm/h)	40 ± 22	44 ± 20	46 ± 21	0.4
Seroconveter (n, %) ^‡^	16 (55)	35 (43)	35 (43)	0.4

* *P *= 0.04, compared to the ≤2 y group

^†^Number of cases that had the duration of fever ≥7 days.

^‡ ^Number of patients who showed negative on the initial examination, and converted to positive on the second examination by Serodia Myco II.

### Clinical and laboratory findings according to pneumonia pattern

The mean age of the bronchopneumonia group was 4.6 ± 2.5 years, significantly younger than that of the segmental/lobar group (6.6 ± 3.0 years; *p *< 0.001). The total duration of fever (*p *= 0.02) and the length of hospitalization (*p *= 0.001) were longer in the segmental/lobar group than in the bronchopneumonia group. Compared with the bronchopneumonia group, the segmental/lobar group recorded lower WBC count (*p *= 0.04), absolute lymphocyte count (1,900 ± 1,300/μL vs. 2,700 ± 1,700/μL, respectively; *p *< 0.001), and platelet count (*p *= 0.02), but higher CRP (2.1 ± 2.3 vs. 5.1 ± 5.3 mg/dL, respectively; *p *< 0.001) (Table 2).

**Table 2:** Clinical and laboratory characteristics for the children with M. pneumoniae pneumonia according to the pneumonia pattern

Group	Bronchopneumonia	Segmental/Lobar	*P*
	(n = 96)	(n = 95)	
Clinical characteristics			
Mean age (y)	4.6 ± 2.5	6.6 ± 3.0	<0.001
Male/Female	47/49	42/53	
Duration of fever (d)	5.5 ± 2.8	6.5 ± 2.8	0.02
Hospitalization (d)	6.4 ± 1.7	7.3 ± 2.3	0.001
			
Laboratory findings			
Haemoglobin (g/dL)	12.3 ± 0.9	12.0 ± 0.9	0.09
Leukocyte (×10^3^//μL)	8.7 ± 4.9	7.4 ± 3.0	0.04
Neutrophil (%)	56 ± 14	65 ± 12	<0.001
Lymphocyte (%)	33 ± 13	25 ± 11	<0.001
Monocyte (%)	9 ± 3	8 ± 3	0.06
Lymphocyte count (/μL)	2700 ± 1700	1900 ± 1300	<0.001
Platelet (×10^3^/μL)	283 ± 98	251 ± 80	0.02
CRP (mg/dL)	2.1 ± 2.3	5.1 ± 5.3	<0.001
ESR (mm/h)	39 ± 18	50 ± 21	0.001
Seroconveter (n,%)	36 (38)	50 (53)	0.04

### Clinical and laboratory findings according to severity of pneumonia

Because patient age may be related to the severity of pneumonia and the levels of laboratory indices (including WBC count and differential), we analyzed the clinical and laboratory parameters of the subgroup of 81 older children aged ≥6 years of age. When we divided and evaluated these school-aged children into three groups according to severity of pneumonia, as previously stated, the patients with more severe pulmonary lesions showed higher CRP levels (*p *= 0.02) and a higher proportion of seroconverters (*p *= 0.001, ANOVA test). The duration of fever and absolute lymphocyte count were significantly different between the bronchopneumonia group and the severe segmental/lobar pneumonia group (*χ*^*2 *^test and independent sample *t*-test) (Table 3).

**Table 3:** Clinical and laboratory findings according to the severity of the pulmonary lesions in the school-aged children (6-14 years of age)

Groups	Bronchopn	Mild	Severe	*P*
	(n = 25)	(n = 33)	(n = 23)	
Clinical characteristics				
Age (y)	8.0 ± 1.9	8.2 ± 2.1	9.1 ± 2.7	0.11
Duration of fever (d)	5.4 ± 2.8	6.0 ± 2.8	7.1 ± 2.6*	0.13
Hospitalization (d)	6.2 ± 1.9	6.7 ± 1.7	7.6 ± 2.4	0.04
				
Laboratory findings				
Leukocyte (×10^3^//μL)	6.9 ± 2.7	7.3 ± 2.3	6.9 ± 2.3	0.8
Neutrophil (%)	60 ± 11	66 ± 9	68 ± 12	0.053
Lymphocyte (%)	28 ± 9	23 ± 7	22 ± 11	0.08
Monocyte (%)	9 ± 4	8 ± 3	8 ± 2	0.5
Lymphocyte count (/μL)	1900 ± 800	1700 ± 700	1400 ± 600*	0.14
CRP (mg/dL)	2.2 ± 1.8	4.6 ± 4.8	5.4 ± 4.3	0.02
Seroconveter (n,%)	4 (16)	16 (49)	15 (65)	0.001

* *P *< 0.05 compared to the bronchopneumonia group

### Clinical and laboratory findings according to diagnostic antibody status

Because the proportion of seroconverters tended to be higher in patients who had more severe pneumonia (Tables 2 and 3), we evaluated the clinical and laboratory findings of the subjects according to diagnostic antibody status: those who were seroconverted (n = 86) and those who had increased titers (n = 105). There were no differences between the two groups in clinical and laboratory indices, except for platelet count (240,000 ± 84,000/μL vs. 289,000 ± 90,000/μL, respectively; *p *< 0.001) (data not shown).

## Discussion

The epidemiologic characteristics of *M. pneumoniae *may differ among populations [8]. Although earlier studies in Western populations reported that the incidence of MP is greatest among school-aged children [1], the age distribution of all patients with MP in the present study was between 9 months and 14 years, with peak incidence at 4-6 years of age (figure 1). We found that MP affects children of all age groups, whereas the clinical phenotype of MP differs with age. Compared with the younger children, the older children had a more severe clinical course, manifested by longer total duration of fever, higher CRP, and a more severe pneumonia pattern. Recent clinical studies have also reported that some clinical features differ between younger children (≤5 years of age) and older children (>6 years of age) [4,5]. Clinical features such as tachypnea, upper respiratory symptoms (coryza), and gastrointestinal symptoms (diarrhea and vomiting) were shown to be more common in younger children [4,5], and the rate of chest radiographic consolidation was higher in older children [5].

Although variation in WBC count is known to be a nonspecific finding in *M. pneumonia *infections, we found that lymphopenia may be one of the characteristics of MP in the acute stage. The mean WBC counts of children with MP at presentation were similar to the normal values for same-age references [14]; however, lymphocyte differential and absolute lymphocyte counts were decreased. In addition, the severity of MP tended to be inversely associated with lymphocyte counts. Studies of adult patients with MP also indicated constant leukopenia with lymphopenia [10,15,16].

Recent clinical studies reported that thrombocytosis was observed in some patients with MP [4,5]. We also found that the platelet counts of patients with MP increased significantly in the convalescent stage, with thrombocytosis (>400,000/μL) observed in 8% of patients at admission but in 33% of patients at discharge. Thus, the degree of platelet counts in MP may be associated with the stage of inflammation and age of the patient. Although this finding may be an epiphenomenon that follows various infections, it is not yet known whether the phenomenon is also observed in viral or other pathogen-induced pneumonias.

Approximately 45% of patients in the present study were seroconverters. This finding verified the observation that many patients are IgM sero-negative at presentation with MP. Absence of diagnostic IgM antibodies in the early stage of systemic infections has been well documented in previous studies of adults and children with MP [17,18] and other infections, including severe acute respiratory syndrome (SARS) due to coronavirus and measles [19,20]. Ozaki *et al. *reported that 31.8% of children with MP were IgM-positive at admission when tested using an EIA (ImmunoCard), but that 88.6% of patients were IgM-positive when tested using paired sera (mean of 8.0 ± 3.0 days apart) [18]. With these findings, because some patients may be false-positives (recent past infection), especially in younger children who may be reservoirs of *M. pneumoniae *during MP epidemics [21,22], the diagnosis of MP based on a single assay for IgM or a PCR without serologic tests is inadequate for patient selection.

Interestingly, among those aged ≥6 years, the group with more severe pneumonia had a greater number of seroconverters; i.e., patients with more severe pulmonary lesions may be more likely to be sero-negative at presentation. Because *M. pneumoniae *infection is controlled by the adaptive immune reaction of the host, including antibodies, patients with severe pneumonia may remain IgM-negative longer in the early stage of MP. Indeed, the three patients in the present series who had the most severe clinical course were seroconverted at the third examination after 1 week.

The detection of cold agglutinins (IgM) is nonspecific for *M. pneumoniae *infections; however, the titer of cold agglutinins in other systemic infections (such as Epstein-Barr virus and adenovirus infections) is rarely ≥1:64, except in *M. pneumoniae *infections [23]. It is reported that detection of cold agglutinins results in a higher sensitivity and specificity for diagnosis of *M. pneumoniae *infections when compared with a serologic test for *M. pneumoniae *[24,25]. According to the patient selection policy employed in this study, nearly all patients among the seroconverters were seroconverted in both serologic assays; among the patients who were seroposive at presentation, 87% showed increased antibody titers ≥4-fold in both serologic assays. Of the 1,083 patients with pneumonia or lower respiratory tract infections during this study period, only 5 showed cold agglutinin changes (≤1:64) without MAA changes. The detection rate of PCR for *M. pneumoniae *in the present study (29%) was lower than those in previous studies of children [26]. We referred our respiratory samples to an external laboratory for PCR and detection of viral antigens. Inconsistent sample delivery times, inadequate dilution of sample volume, and other undetermined factors may (in part) have affected our results.

It is recently reported that co-infection of *M. pneumoniae *with other bacterial and/or viral pathogens is not rare [2,27,28]. In the present study, two cases of *M. pneumoniae *pneumonia were co-infected with viral infections and there were no positive cases of bacterial blood culture. Because we did not perform extensive microbiological testing of all the subjects, we cannot exclude the possibility that some children might have had co-infection with other bacterial or viral pathogens. However, it is assumed that our methodology (two serologic tests and two examination times) would have reduced patient-selection bias as much as possible. The clinical implications of mixed infections, compared with a sole agent, remain unresolved [28].

All children in this study were treated with amoxicillin with clavulanate and a macrolide (clarithromycin or roxithromycin); 75% of the patients defervesced within 2 days and 83% of patients defervesced within 3 days after initiation of antibiotic treatment. Of the patients with a fever duration >4 days, 14 who were non-responsive to antibiotics and had progressive pneumonia received additional prednisolone treatment (1 mg/kg/day for 3-4 days, tapering within 1 week); these patients showed rapid improvement of clinical and radiographic findings, as previously observed [12]. The beneficial effects of systemic corticosteroids on severe or fatal MP have been well documented in children, adults, and experimental animals [10-13,29]. A recent article reviewed clinical, experimental, and pathologic studies for the notion of a host immune response including cell-mediated immunity in *M. pneumonia *infections [8].

Because the present study was performed during a recent nationwide epidemic and the subjects were all inpatients, our results might not reflect the exact epidemiologic characteristics of *M. pneumonia *infections. Clinicians can encounter intermittent endemic cases prior to an epidemic with a 3-5 yr cycle, and our results may help to prepare for coming MP epidemics and to understand the clinical characteristics of MP.

## Conclusion

The clinical phenotype of MP differs with age, with a longer period of fever, higher CRP, and more severe pulmonary lesions observed in older children. The severity of pulmonary lesions was associated with lower lymphocyte count and higher sero-negativity of diagnostic IgM antibodies at presentation. Short-term paired IgM serologic test in the acute stage may assist in obtaining an early and definitive diagnosis of MP and reduce bias in patient selection. Further studies are required into the pathogenesis of *M. pneumoniae *infection.

## Abbreviations

CRP: C-reactive protein; MAA: microparticle agglutinin assay; MP: *Mycoplasma pneumoniae *pneumonia; PCR: polymerase chain reaction; WBC: white blood cell

## Competing interests

The authors declare that they have no competing interests.

## Authors' contributions

All authors read and approved the final manuscript. KYL had primary responsibility for the study concept and design, and writing the manuscript. YSY participated in preliminary data collection, data analysis, and writing the manuscript. JYH and JUR participated in patient care, data collection, and data analysis. JHK contributed to interpretation of the data and editing of the manuscript. JSL supervised the design and execution of the study. JCK read the chest radiographs.

## Pre-publication history

The pre-publication history for this paper can be accessed here:

http://www.biomedcentral.com/1471-2431/10/48/prepub
